# Filovirus Research in Gabon and Equatorial Africa: The Experience of a Research Center in the Heart of Africa

**DOI:** 10.3390/v4091592

**Published:** 2012-09-13

**Authors:** Eric Leroy, Jean Paul Gonzalez

**Affiliations:** 1 Centre International de Recherches Médicales de Franceville (Franceville International Center for Medical Research), CIRMF, Libreville BP 2105, Gabon; Email: jean-paul.gonzalez@ird.fr; 2 Institut de Recherche pour le Développement, IRD, Marseille 13055, France; 3 French Ministry of Foreign and European Affairs, French Embassy 75116, Gabon

**Keywords:** filovirus, Central Africa, CIRMF, Gabon, bats, immunology, Ebola, hemorrhagic fever

## Abstract

Health research programs targeting the population of Gabon and Equatorial Africa at the International Center for Medical Research in Franceville (CIRMF), Gabon, have evolved during the years since its inception in 1979 in accordance with emerging diseases. Since the reemergence of Ebola virus in Central Africa, the CIRMF “Emerging Viral Disease Unit” developed diagnostic tools and epidemiologic strategies and transfers of such technology to support the response of the National Public Health System and the World Health Organization to epidemics of Ebola virus disease. The Unit carries out a unique investigation program on the natural history of the filoviruses, emergence of epidemics, and Ebola virus pathogenesis. In addition, academic training is provided at all levels to regional and international students covering emerging conditions (host factors, molecular biology, genetics) that favor the spread of viral diseases.

## 1. Development of Research Facilities for the Study of Viral Hemorrhagic Fevers

**Figure 1:**
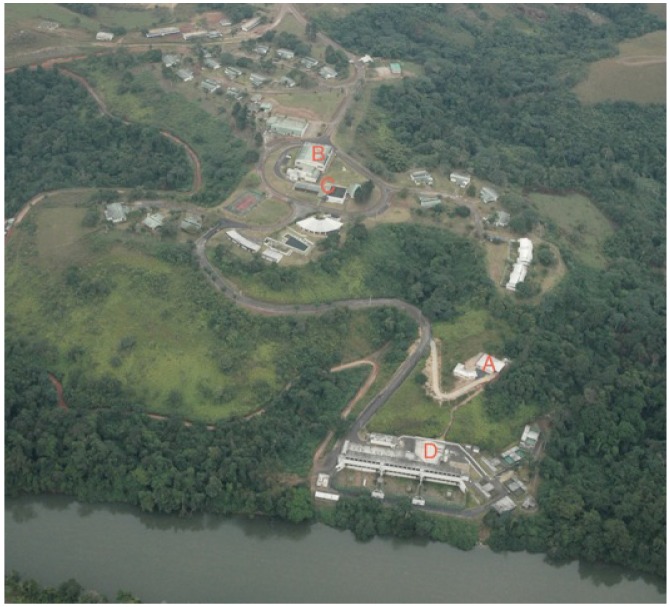
The International Centre for Medical Research of Franceville (CIRMF). Campus aerial view: the P4 laboratory: **A** = P4 laboratory facilities; **B** = Main Building; **C** = P3 Laboratory; **D** = Primatology Center.

The International Centre for Medical Researches of Franceville (CIRMF) was founded in 1974 by His Excellency El Hadj Omar Bongo Ondimba, President of the Gabonese Republic, and Mr. Pierre Guillaumat, the chairman of the petroleum company, Total Gabon. The Centre was inaugurated on December 5th, 1979 with the participation of numerous internationally eminent scientists (Figure 1).

**Figure 2:**
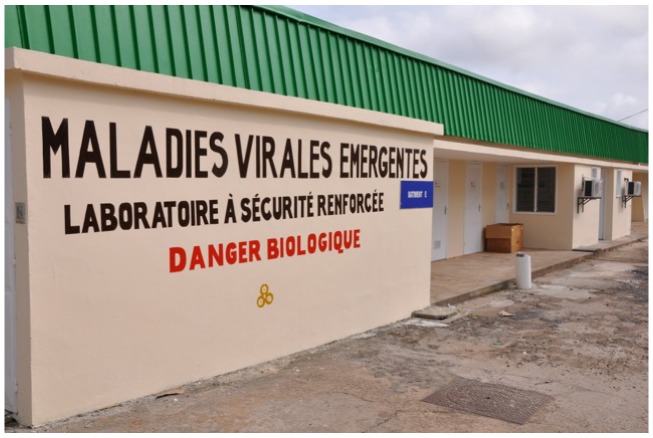
The International Centre for Medical Research of Franceville (CIRMF). Emerging Viral Diseases Unit P3 laboratory.

**Figure 3:**
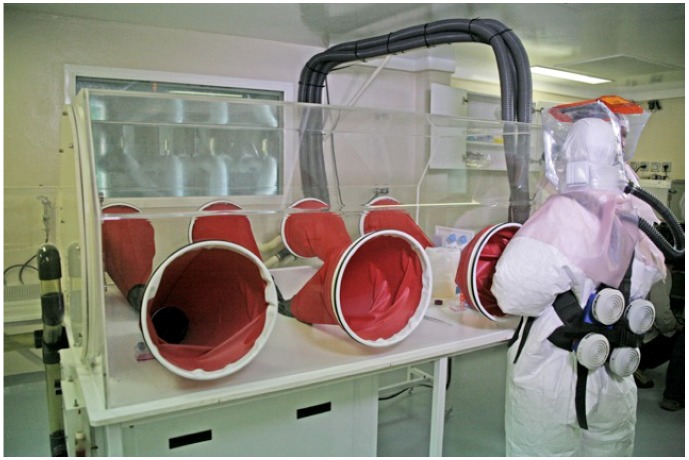
The Present CIRMF High Security P4 Laboratory with “Glove Box”.

In the 1990s, viral hemorrhagic fevers became a focus of attention in Equatorial. The decision to develop a high security laboratory for the study of Ebola virus disease came after a 1996 Ebola virus disease outbreak in Mayibout area, Gabon. The main objective was to develop the potential for rapid and specific diagnosis on viral hemorrhagic fevers and to have a backup for field investigation of severe viral hemorrhagic fever epidemics. Because the unique expertise and interaction of the CIRMF team along with the international World Health Organization (WHO) teams for Ebola virus disease, the Gabonese government agreed to such a project. The first BSL3+ (including negative pressure and glove box) laboratory was built in 1997, mostly financed by the Foreign Ministry of France (Figure 2). This laboratory was built in a period of quasi “emergence” of successive Ebola virus disease outbreaks in Gabon, and the plans to upgrade the BSL3+ laboratory were not efficient. A specific research unit was founded in 1998 to study emerging infectious diseases: The Emerging Viral Disease Unit (UMVE). Thanks to work done between 1996 and 1998, both in the field and in research, CIRMF became a National Reference Laboratory and a WHO Collaborating Center in the Equatorial African region. A second high security laboratory for Risk Group 3/4 Agents, mostly funded by the TOTAL Gabon oil company and the Gabonese Government, was built between 2003 and 2008 on CIRMF campus (Figure 3). This BSL-4 laboratory was commissioned by a combined team of experts from the Pasteur Institute of Paris, National Institute of Health and Medical Research, France, and Jean Mérieux P4 laboratory of Lyon. 

## 2. Infrastucture

### 2.1. Facilities

On 45 hectares, the CIRMF campus has a working space of 2,500 square meters composed of a main building, laboratories, service buildings, and living accommodations (Figure 1). The present high containment and high security laboratory, operated by the Emerging Viral Diseases Unit (UMVE), is one of 2 laboratories in Africa that can manipulate Risk Group 3/4 Agents (*i.e.*, Ebola, Marburg, and Crimean-Congo hemorrhagic fever viruses). Research, including isolation and characterization of these highly pathogenic viruses is performed in accordance with international rules defined by WHO on the handling Risk Group 3/4 Agents Updated equipment includes a double door autoclave, thermo regulated cabinet, a high security centrifuge system, a conventional photonic microscope, a virus isolation unit, and two independent rooms the can be shut down alternatively after decontamination when necessary. An uninterruptable controlled electrical supply for refrigeration, computer systems, and other systems is ensured by two back-up power plants. The telecommunication network consists of mobile phones and the Internet through a dedicated satellite antenna. Other service units consist of a Primate Center, the Gorilla and Chimpanzee Study Station in Lopé National Park, and the Dienga Health Observatory. Investigators from this observatory conduct field studies on the prevalence of viral and parasitic diseases and their implications for public health [1]

Dedicated to medical research, the Primate Center houses more than 450 primates belonging to ten different African species (e.g., chimpanzees (*Pan troglodytes*), gorillas (*Gorilla sp*), mandrills (*Mandrillus sphinx*), guenons (*Cercopithecus sp.*), collared mangabeys (*Cercocebus torquatus*), greater spot-nosed monkeys (*Cercopithecus nictitans*), vervet monkeys (*Chlorocebus pygerythrus*) and an Asian macaque (*Macaca sp.*) colony. One of the largest primate centers in Africa, the Primate Center is equipped with level A2 and A3 animal facilities for scientific research protocols. The Great Apes are housed in large open-air facilities. Semi-free living colony of twelve forested hectares harbor about half of the primates at the Primate center. At the Gorillas and Chimpanzee Study Station, researchers study ecological approaches to the emergence of zoonotic diseases, inter-species transmission of pathogens, and disease outbreaks in humans and wild animals [2]. 

### 2.2. Organization

Several councils composed of eminent scientists and politicians participate in the functioning of CIRMF. The Administrative Council, created by the two cofounders of CIRMF, the Gabonese Government and Total-Gabon is open to other partners like the French Foreign Office, the Institute for Research Development (IRD), Marseille, and other European agencies. The Scientific Council presided by Professor Patrice Debré (National Institute of Health and Medical Research, France) recommends scientific policy to the Board of Directors [2].

### 2.3. Funding

Running costs are funded by the Ministry of Economy, Gabon, the national petroleum company of Total-Gabon, and the Ministry of Foreign and European Affairs, France. Several international agencies participate in a variety of financial supports including scientists’ salaries, equipment, research projects, and academic grants (e.g., IRD, WHO, United States Agency for International Development, National Center for Scientific Research, France).

## 3. Objectives: Training, Public Health Support, and Research

### 3.1. Training

Technical laboratory training support of Gabonese teams and other African countries has been one of the major aims of CIRMF. The UMVE actively participates in the academic training at the Regional Graduate School and the different State universities of Equatorial Africa. A special relationship with the “Health Sciences University” of Libreville and the “Sciences and Technology University” of Masuku in Franceville encourages collaborative research projects with teachers and supports students of the Faculties of Medicine and Sciences in the preparation of doctoral theses. CIRMF receives doctoral and post-doctoral scientists from other universities of developed countries (e.g., Bonn, Marseille, Montpellier, and Tübingen Universities). Continuing medical education in the form of post-doctoral workshops are held at the CIRMF for discussion and demonstration of modern techniques. 

### 3.2. Public Health Support through National, Regional, and International Partnerships

#### 3.2.1. National Partnerships

As a National reference laboratory, CIRMF has the following roles: diagnosis of suspected cases during outbreaks of viral hemorrhagic fevers or severe clinical infectious syndromes; development of new methods for diagnosing such infections; surveillance of animal fatalities in reservoir or intermediate hosts; and intervention during outbreaks of unknown etiology. CIRMF diagnosed infections of more than 70 pathogens that could not be identified in other biology laboratories throughout the country. CIRMF maintains close ties to several components of the National Healthcare system, such as Amissa Bongo Regional Hospital in Franceville or the Sino-Gabonese Friendship Hospital. 

In order to facilitate national and international scientific exchanges including scientists, equipment, biological specimens, the capital of Gabon, Libreville, is part of CIRMF operational system. Hosted by the University of Health Sciences, Libreville, one laboratory is now operational. Tight connections with other scientific teams in Libreville are under development (*i.e.*,: Military Hospital, Libreville; General Hospital, Libreville; A. Schweitzer Lambarene Foundation).

#### 3.2.2. Regional Partnerships

The Emerging Viral Diseases Unit, CIRMF, proposes forming a research partnership to study infectious diseases transmitted by animals of the tropical rain forests regions of Equatorial Africa. The proposed partnership builds on such existing collaborations of several years between the major research centers of 2 other French speaking Equatorial African countries, namely the National Public Health Laboratory in Brazzaville, Republic of the Congo, and the Institute for Biomedical Research in Kinshasa, Democratic Republic of Congo. An international partnership with the IRD, Marseille, France, and the Institute of Virology, Bonn, Germany, will assist in the development of this regional partnership. 

#### 3.2.3. International Partnerships

With the WHO Regional Office, Brazzaville, Republic of the Congo, the UMVE-CIRMF field team participates along with other international partners (e.g., Centers for Disease Control and Prevention, USA; Laboratory Centre for Disease Control and National Microbiology Laboratory, Winnipeg, Canada; P4 Laboratory of the National Institute for Communicable Diseases, South Africa) to respond to all Ebola virus disease epidemics in Africa. CIRMF aims to use laboratory and field expertise be a regional focal resource in conjunction with local health authorities to organize epidemic responses. CIRMF expertise is also offered from by entering into laboratory-to-laboratory agreements. Also, an informal international laboratory network for the diagnosis and surveillance of severe infectious clinical syndromes includes: the Institut National de Recherche Biomédicale, Democratic Republic of the Congo; Laboratoire National de Santé Publique de Brazzaville, Republic of the Congo; Metabiota/Laboratoire des Maladies Emergentes, Yaoundé, Cameroon; Pasteur Institute, Bangui, Central African Republic; Institute of Virology, Bonn University, Germany; and P4 Jean Mérieux Lyon, France. Exchange of materials, equipment, and personnel is facilitated through memorandums of understanding. 

CIRMF holds more than 250,000 samples of various origins in a biological repository, which is accessible to the International scientific community. The UVME assists the National Public Health System in consolidating and formalizing microbiological monitoring of the Equatorial African sub-region. Ultimately, CIRMF will be positioned as a Center of Excellence for microbiological surveillance and research in a global network. 

### 3.3. Research from the Emerging Viral Disease Unit

#### 3.3.1. Developing Tools and Methods for Virus Detection

Developing diagnostic tools and strategies is the main driver to improve surveillance and research of emerging viral diseases. A strategic choice was made to link syndromes to an etiological agent, including hemorrhagic syndromes. To isolate and diagnose highly pathogenic viruses, a progressive and diversified methodology was applied. The first approach used real-time virus-specific PCR (qRT-PCR). If the first approach was not successful, conventional RT-PCR was implemented using degenerate consensus primers targeting conserved regions of the genome. Ultimately, random amplification of nucleotide sequences was directly applied to the original biological material (DNA chip re-sequencing, meta-genomic pyrosequencing (454 Life Sciences, Branford, CT)).

#### 3.3.2. Understanding Filovirus Natural History

**Figure 4:**
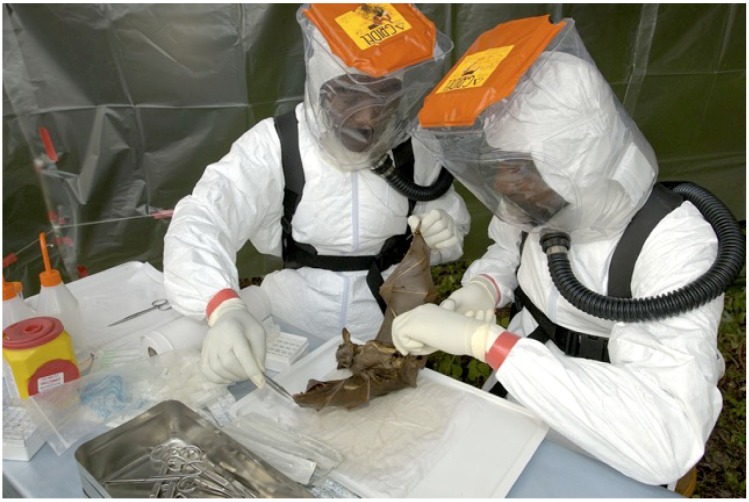
Field Biosafety and Trapping Potential Ebola Virus Reservoir Bats in Gabon.

UMVE studied the factors implicated in the three steps that led to Ebola virus and Marburg virus diseases emergence in humans. These steps include: the identification of reservoir species, the circulation within the natural host, the crossing to intermediary animal species, and finally the direct transmission to humans from great apes and fruit bats. Antibodies and nucleotide sequences specific for Ebola virus were detected in the liver and spleen of fruit bat belonging to three species (*Hypsignathus monstrosus*, *Epomops franqueti*, *Myonycteris torquata*) in Gabon and Republic of the Congo (Figure 4). Antibodies and nucleotide sequences specific for Marburg virus were found in the Egyptian fruit bat (*Rousettus aegyptiacus*) in Gabon, suggesting that bats might be reservoirs for filoviruses [3,4,5]. 

We showed that Ebola virus caused extensive epizootics among gorillas and chimpanzees, killing thousands of animals during the last decade in parts of Gabon and Republic of the Congo [4]. We characterized the viral variants associated with all Ebola virus disease outbreaks that occurred between 1996 and 2008 and developed new epidemiological models of Ebola virus disease epidemics, based on the identification of several independent epidemic chains. The identification of multiple variants during the 2001 Gabon/Republic of Congo outbreak and two phylogenetically divergent lineages suggest independent introductions into great ape and human populations following multiple viral spillovers from a reservoir host [6,7]. In this “multi-emergence” hypothesis, Ebola virus disease outbreaks would occur episodically during certain ecological conditions caused by habitat disturbances or climatic phenomena. This hypothesis also implicitly assumes that Ebola virus was present in Equatorial Africa long before the first documented disease outbreak in 1976, as supported by various serological surveys. Furthermore, we recently showed that the 2007 Luebo outbreak in the Democratic Republic of the Congo was linked to massive fruit bat migration, strongly suggesting that humans could be infected directly by bats or by consumption of bats [8]. 

#### 3.3.3. Ebola Virus Disease Pathogenesis

In the study of immunological mechanisms of Ebola virus disease humans, we showed that fatal infection is associated with aberrant innate immunity and global suppression of adaptive immunity [9,10,11,12]. The innate immune reaction is characterized by a ‘cytokine storm’, with a hyper secretion of numerous pro-inflammatory cytokines, chemokines and growth factors, and by the noteworthy absence of antiviral interferon (IFN)-α [9,13,14]. Immunosuppression of adaptive immunity is characterized by very low levels of circulating cytokines produced by T lymphocytes and by massive loss of peripheral CD4 and CD8 lymphocytes, probably through Fas/FasL-mediated apoptosis. Finally, we hypothesized that a viral protein with super-antigen activity might be involved in the massive T cell apoptosis [15]. In striking contrast with fatal outcome, effective control of Ebola virus infection is associated with balanced immune responses in survivors. Asymptomatic Ebola virus infection was demonstrated in humans during the 1996-97 disease outbreak in Gabon [16]. Asymptomatic infection was associated with an early strong inflammatory response that may be involved in the early inhibition of viral replication [16,17]. Consistent with this discovery, we showed a decade later that a large fraction of the human population living in forested areas of Gabon has both humoral and cellular immunity to Ebola virus [18]. In the absence of identified risk factors, the high prevalence of ‘immune’ individuals suggests a common source of human exposure such as fruits contaminated by bat saliva.

Initially focused on Ebola virus disease, UMVE science policy was redirected since 2007 by expanding the main research themes to other emerging viral diseases that could threaten public health in the Congo basin (Table 1) [19,20,21,22]. 

#### 3.3.4. Challenges of Conducting Filovirus Research in Gabon

CIRMF is geographically isolated from the capital of Gabon, Libreville. The Libreville office is essential to the Franceville headquarters as it coordinates visits from staff on field missions, and forwards imported equipment to headquarters. The capital is accessible by a 12-hour ride in a four-wheel drive vehicle or in a train (three times/week schedule) covering 641 km. Due to tropical weather, four weekly domestic plane rotations often fly on an inconsistent schedule. Ultimately CIRMF needs to be largely autonomous in term of electrical power (*i.e.*,: unexpected fuel supply disruption), cold chain with the necessity to maintain *in situ* a unit of liquid nitrogen production (repository), and purified water supply.

**Table 1:** Viruses identified by the Emerging Viral Unit using CIRMF high containment facilities.

Family	Genus/Virus *	Origin/Country **	Year	Host	Genetics/virus isolates ***
*Filoviridae*	*Ebolavirus*/Ebola virus ****	Mayibout 2/Gabon	1996	Human	+/+
	*Ebolavirus*/Ebola virus	Booué/Gabon	1997	Human	+/+
	*Ebolavirus*/Ebola virus	Mendemba/Gabon	2001	Human	+/+
	*Ebolavirus*/Ebola virus	Makokou/Gabon	2002	Human	+/+
	*Ebolavirus*/Ebola virus	Ilahounéné/Gabon	2002	Human	+/+
	*Ebolavirus*/Ebola virus	Ekatangaye/Gabon	2002	Human	+/+
	*Ebolavirus*/Ebola virus	Ekata/Gabon	2001	Human	+/+
	*Ebolavirus*/Ebola virus	Olloba/Gabon	2001	Human	+/+
	*Ebolavirus*/Ebola virus	Mbomo/Gabon	2002	Human	+/+
	*Ebolavirus*/Ebola virus	Yembelengoye/Gabon	2003	Human	+/+
	*Ebolavirus*/Ebola virus	Mvoula/RC *	2003	Human	+/+
	*Ebolavirus*/Ebola virus	Mbandza/RC	2003	Human	+/+
	*Ebolavirus*/Ebola virus	Etoumbi/RC	2005	Human	+/+
	*Ebolavirus*/Ebola virus	Luebo/RDC	2007	Human	+/+
	*Ebolavirus*/Ebola virus	Luebo/RDC	2008	Human	+/+
	*Ebolavirus*/Ebola virus	Ekata/Gabon	2002	Bat	+/-
	*Ebolavirus*/Ebola virus	/Gabon	2002	Chimp	+/-
	*Ebolavirus*/Ebola virus	/Gabon	2002	Gorilla	+/-
	*Ebolavirus*/Ebola virus	/Gabon	2002	Duiker	+/-
	*Ebolavirus*/Ebola virus	/Gabon	2003	Chimp	+/-
	*Ebolavirus*/Ebola virus	/Gabon	2003	Gorilla	+/-
	*Ebolavirus*/Ebola virus	/RC	2005	Chimp	+/-
	*Ebolavirus*/Ebola virus	/RC	2005	Gorilla	+/-
	*Marburgvirus*/Marburg virus	Lambaréné/Gabon	2005	Bat	+/-
	*Marburgvirus*/Marburg virus	Tchibanga/Gabon	2006	Bat	+/-
	*Marburgvirus*/Marburg virus	Makokou/Gabon	2009	Bat	+/-
*Togaviridae*	*Alphavirus*/Chikungunya virus	Malabo/EG	2007	Human	+/-
	*Alphavirus*/Chikungunya virus	Libreville/Gabon	2007–2008	Human	+/+
	*Alphavirus*/Chikungunya virus	Oyem/Gabon	2007	Human	+/+
	*Alphavirus*/Chikungunya virus	Lambaréné/Gabon	2008–2009	Human	+/+
	*Alphavirus*/Chikungunya virus	Ndjolé/Gabon	2008	Human	+/+
	*Alphavirus*/Chikungunya virus	Lastourville/Gabon	2007	Human	+/+
	*Alphavirus*/Chikungunya virus	Franceville/Gabon	2010	Human	+/+
	*Alphavirus*/Chikungunya virus	/Gabon	2007–2010	Human	+/+
	*Alphavirus*/Chikungunya virus	Brazzaville/RC	2011	Human	+/+
	*Alphavirus*/Chikungunya virus	/RDC	2010–2011	Human	+/-
	*Alphavirus*/Chikungunya virus	/Gabon	2007–2010	Mosquito	+/-
*Flaviviridae*	*Flavivirus*/Dengue virus 2	Libreville/Gabon	20072008	Human	+/+
	*Flavivirus*/Dengue virus 2	Oyem/Gabon	2007	Human	+/+
	*Flavivirus*/Dengue virus 2	Lambaréné/Gabon	2008–2009	Human	+/+
	*Flavivirus*/Dengue virus 2	Ndjolé/Gabon	2008	Human	+/+
	*Flavivirus*/Dengue virus 2	Lastourville/Gabon	2007	Human	+/+
	*Flavivirus*/Dengue virus 2	Franceville/Gabon	2010	Human	+/+
	*Flavivirus*/Dengue virus 2	/RDC	2011	Human	+/-
	*Flavivirus*/Dengue virus 2	/Gabon	2007–2010	Mosquito	+/-
	*Flavivirus*/Dengue virus 1	Libreville/Gabon	2007–2008	Human	+/+
	*Flavivirus*/Dengue virus 1	Lambaréné/Gabon	2008–2009	Human	+/+
	*Flavivirus*/Dengue virus 1	Franceville/Gabon	2010	Human	+/+
	*Flavivirus*/Dengue virus 1	/RDC	2011	Human	+/-
	*Flavivirus*/Dengue virus 3	Franceville/Gabon	2010	Human	+/+
*Flaviviridae*	*Flavivirus*/Zika virus	Libreville/Gabon	2007	Human/Mosquito	+/-
	*Flavivirus*/Wesselsbron virus	/Gabon	2010	Duiker	+/-
	*Flavivirus*/Spondweni virus	/Gabon	2010	Duiker	+/-
*Bunyaviridae*	*Nairovirus*/CCHFV	/RDC	2009	Human	+/-
	*Phlebovirus*/RVFV *	/Gabon	2011	Tick	+/-
*Picornaviridae*	Enterovirus/PV1	Pointe Noire/RC	2010	Human	+/+
	Rhinovirus/UT *****	/Gabon	2010–2012	Human	+/-
	Parechovirus/UT	/Gabon	2010–2012	Human	+/-
	Parechovirus/UT	/Gabon	2010–2012	Human	+/-
	Enterovirus/UT	/Gabon	2011	Monkeys	+/-
*Coronaviridae*	*Coronavirus*/HCoV NL63	/Gabon	2010–2012	Human	+/-
	*Coronavirus*/HCoV HKU1	/Gabon	2010–2012	Human	+/-
	*Coronavirus*/HCoV OC43	/Gabon	2010–2012	Human	+/-
	*Coronavirus*/HCoV 229E	/Gabon	2010–2012	Human	+/-
	*Coronavirus*/*UT*	/Gabon	2005–2009	Bat	+/-
*Paramyxoviridae*	*Pneumovirus*/hRSV	/Gabon	2010–2012	Human	+/-
	*Respirovirus*/PIV 1	/Gabon	2010–2012	Human	+/-
	*Rubulavirus*/PIV 2	/Gabon	2010–2012	Human	+/-
	*Respirovirus*/PIV 3	/Gabon	2010–2012	Human	+/-
	*Rubulavirus*/PIV 4	/Gabon	2010–2012	Human	+/-
	*Metapneumovirus*/hMPV	/Gabon	2010–2012	Human	+/-
	*Rubulavirus*/*UT*	/Gabon	2005–2009	Bat	+/-
	*Morbillivirus*/*UT*	/Gabon	2005–2009	Bat	+/-
	*Henipavirus*/*UT*	/Gabon	2005–2009	Bat	+/-
*Orthomyxoviridae*	*Influenzavirus A*/FLUAV H1N1	/Gabon	2010–2012	Human	+/-
	*Influenzavirus A*	/Gabon	2010–2012	Human	+/-
	*Influenzavirus B*	/Gabon	2010–2012	Human	+/-
*Adenoviridae*	*Adenovirus*/*UT*	/Gabon	2010–2012	Human	+/-
*Reoviridae*	*Rotavirus*/*UT*	/Gabon	2010–2012	Human	+/-
*Caliciviridae*	*Sapovirus*/*UT*	/Gabon	2010–2012	Human	+/-
	*Norovirus*/*NoVG2/2*	/Gabon	2010–2012	Human	+/-
	*Norovirus*/*NoV1/2*	/Gabon	2010–2012	Human	+/-
*Astroviridae*	*Astrovirus*/*Human astrovirus*	/Gabon	2010–2012	Human	+/-
*Herpesviridae*	*Simplexvirus*/HSV1 & HSV2	/Gabon	2010–2012	Human	+/-
	*Simplexvirus*/HSV1 & HSV2	/Gabon	2010–2011	Human	+/-
	*Varicellovirus*/VZV	/Gabon	2010–2012	Human	+/-
	*Varicellovirus*/VZV	/Gabon	2010–2011	Human	+/-
	*Cytomegalovirus*/CMV	/Gabon	2010–2012	Human	+/-
	*Cytomegalovirus*/CMV	/Gabon	2010–2011	Human	+/-
	*Roseolovirus*/HHV6	/Gabon	2010–2012	Human	+/-

* Each line refers to a serial virus identification and/or isolation (not to one single strain). ** RC = Republic of the Congo, DRC = Democratic Republic of the Congo; EG = Equatorial Guinea. *** Genetic characterization by partial or total sequencing/Virus isolation on cell culture; all Ebola virus strains are of the “Ebola-Zaïre” type; **** Undetermined Type.

## 4. Conclusions

CIRMF is uniquely suited to study infectious diseases of the Congolese tropical rain forest, the second world’s largest rain forest. As a central point of a North-South transect of the rain forest, the Center is able to study the biodiversity of Africa including animal species, microbes, and parasites. 

CIRMF is dedicated to conduct medical research of the highest standard, and is the only facility of its type in Equatorial Africa. With unrivalled infrastructure, multiple sites, and multidisciplinary teams, the Center promotes a modern healthcare system in Gabon. CIRMF teams are engaged in trans-disciplinary projects bringing together specialists from the health sciences, biological sciences, veterinary medicine, conservation, the humanities, and environmental sciences. The Center welcomes partnerships from around the world to work on global human health issues. 
